# Vaping-Induced Proteolysis Causes Airway Surface Dehydration

**DOI:** 10.3390/ijms242015348

**Published:** 2023-10-19

**Authors:** Arunava Ghosh, Raymond D. Coakley, Neil E. Alexis, Robert Tarran

**Affiliations:** 1 Department of Cell Biology & Physiology, University of North Carolina at Chapel Hill, Chapel Hill, NC 27514, USA; arunava_ghosh@med.unc.edu; 2 Marsico Lung Institute, University of North Carolina at Chapel Hill, Chapel Hill, NC 27514, USA; ray_coakley@med.unc.edu; 3 Center for Environmental Medicine, Asthma and Lung Biology, University of North Carolina at Chapel Hill, Chapel Hill, NC 27514, USA; neil_alexis@med.unc.edu; 4 Division of Genetic, Environmental and Inhalational Disease, Department of Internal Medicine, Kansas University Medical Center, Kansas City, KS 66103, USA

**Keywords:** e-cigarettes, ENaC, airway surface liquid, bronchoalveolar lavage fluid, elastase, menthol

## Abstract

Proteases such as neutrophil elastase cleave and activate the epithelial sodium channel (ENaC), causing airway dehydration. Our current study explores the impact of increased protease levels in vapers’ airways on ENaC activity and airway dehydration. Human bronchial epithelial cultures (HBECs) were exposed to bronchoalveolar lavage fluid (BALF) from non-smokers, smokers and vapers. Airway surface liquid (ASL) height was measured by confocal microscopy as a marker of hydration. ENaC cleavage was measured by Western blotting. Human peripheral blood neutrophils were treated with a menthol-flavored e-liquid (Juul), and the resulting secretions were added to HBECs. BALF from smokers and vapers significantly and equally increased ENaC activity and decreased ASL height. The ASL height decrease was attenuated by protease inhibitors. Non-smokers’ BALF had no effect on ENaC or ASL height. BALF from smokers and vapers, but not non-smokers, induced ENaC cleavage. E-liquid-treated neutrophil secretions cleaved ENaC and decreased ASL height. Our study demonstrated that elevated protease levels in vapers’ airways have functional significance since they can activate ENaC, resulting in airway dehydration. Lung dehydration contributes to diseases like cystic fibrosis (CF), chronic obstructive pulmonary disease (COPD) and asthma. Thus, our data predict that vaping, like smoking, will cause airway surface dehydration that likely leads to lung disease.

## 1. Introduction

Electronic cigarettes (e-cigarettes) are battery-powered devices that deliver nicotine and flavors in a propylene glycol/vegetable glycerin vehicle. In the EU, nicotine content in e-cigarettes is regulated at 3%, but in the USA, it is unregulated and is sold at ≥6% [1]. E-cigarettes have been proposed as smoking cessation aids [2] and as a method of harm reduction compared to combustible cigarettes [3]. However, despite being touted as a safer alternative to cigarettes, no long-term safety data are available. Over the last decade, e-cigarette use has increased, especially in the USA and EU [4,5]. In the UK, where e-cigarettes are favorably promoted, this number appears to have stabilized, with ~7% of the population being regular users. Importantly, previous and current use of e-cigarettes have been associated with respiratory symptoms, including asthma, chronic bronchitis, emphysema and wheezing [6,7,8]. Moreover, a retrospective study found that symptoms worsened after patients switched to vaping [9].

COPD is a chronic and progressive inflammatory condition encompassing emphysema and chronic bronchitis that leads to irreversible airflow obstruction. COPD is one of the leading causes of death worldwide [10] and is associated with the chronic inhalation of noxious gasses such as combustible cigarette smoke. Mucus dehydration, caused by altered salt and water homeostasis in parallel with mucin hypersecretion, is a key feature of COPD [11]. Here, airway dehydration reduces mucus transport, leading to mucus stasis, plugging and inflammation, all of which obstruct airflow. Indeed, mucus dehydration inversely correlates with forced expiratory volume in 1 s (FEV_1_) in COPD patients and mucus plugging is a predictor of COPD mortality [12,13]. Two apical membrane ion channels, namely the cystic fibrosis transmembrane conductance regulator (CFTR) Cl^−^ channel and the epithelial Na^+^ channel (ENaC), play pivotal roles in regulating airway surface hydration by driving fluid secretion and absorption, respectively [11,14]. ENaC is a heterotrimer of α- β- and γ- subunits that combine to form the Na^+^-selective channel. In COPD lungs, expression of α- and β-ENaC subunits inversely correlates with lung function [15]. Similarly, increased ENaC activity caused by β-ENaC overexpression in mice results in lung disease that is similar to what is seen in COPD patients, including airway dehydration, neutrophilic inflammation, decreased mucus transport, mucus obstruction, airspace enlargement and increased mortality [16,17].

The critical roles played by proteases in COPD pathogenesis are well-described and include facilitating airway remodeling and destruction of both the basement membranes and alveolar walls [18]. ENaC must be proteolytically cleaved (especially γ-ENaC) by proteases such as neutrophil elastase in order to activate and conduct Na^+^ [19,20]. We and others have previously reported that chronic e-cigarette use affects the innate defense mechanisms of the lungs and causes protease release from inflammatory cells [21,22]. Since elevated neutrophil elastase levels directly activate ENaC, it is important to understand its potential impact on airway hydration. Indeed, in vitro models have demonstrated that neutrophil elastase exposure to well-differentiated HBECs drives ASL dehydration by activating ENaC [23]. We therefore tested the hypothesis that elevated protease levels in vapers’ BALF could activate Na^+^ absorption and drive ASL dehydration.

## 2. Results

### 2.1. Smokers’ and Vapers’ BALF Alter ENaC Subunit Composition

**Figure 1:**
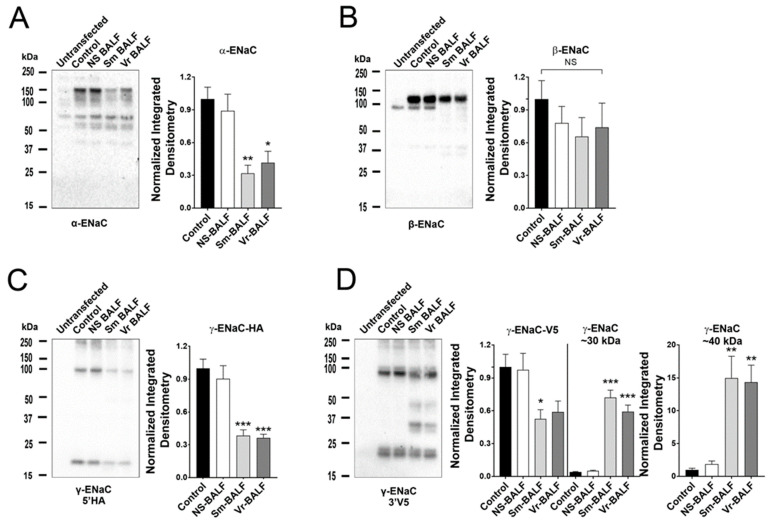
Vapers’ and smokers’ BALF supernatant alters ENaC subunit expression. HEK293T cells were transfected with α-gfp, β, HA-γ-V5 ENaC subunits and treated with concentrated BALF supernatants from non-smokers, smokers and vapers for 1 h. The impact of BALF supernatant exposure on ENaC subunits is shown for (**A**) α-, (**B**) β-, (**C**) 5′ HA-γ and (**D**) 3′ V5- γ-ENaC, with bar graphs of corresponding integrated densitometry. * = *p* < 0.05, ** = *p* < 0.01, *** = *p* < 0.001 different to control. All n = 6 per group. All data were analyzed using the Kruskal–Wallis test followed by Dunn’s multiple comparison post-test.

ENaC is cleaved and activated by a large variety of proteases, including neutrophil elastase [24]. Since this protease was increased in smokers’ and vapers’ lungs [21,22], we first validated that we could detect ENaC subunit cleavage following neutrophil elastase exposure. This protease induced cleavage of α-ENaC, leading to the emergence of a ~40 kDa cleavage fragment (Supplementary Figure S1A). Neutrophil elastase also cleaved β-ENaC and induced the formation of two fragments at ~30 and ~22 kDa (Supplementary Figure S1B). Similarly, we detected cleavage of γENaC (Supplementary Figure S1C,D). We then added pooled BALF supernatants from age- and sex-matched non-smokers (who were also non-vapers), smokers and vapers to HEK293T cells expressing α-gfp, β, HA-γ-V5 ENaC for 1 h, after which time cells were lysed and prepared for Western blotting. Representative blots for ENaC and the GAPDH loading control are presented in Figure 1 and Figure S2A, respectively. Unlike BALF from non-smokers, BALF from vapers and smokers significantly reduced the size of full-length α-ENaC (Figure 1A). Consistent with previous reports that β-ENaC is less important for ENaC activation than the other subunits [20], no significant reduction of full-length β-ENaC was observed (Figure 1B). γ-ENaC cleavage was examined by probing for antibodies against both 5-prime HA and 3-prime V5 epitopes. Both vapers’ and smokers’ BALFs caused significant decreases in full-length γ-ENaC protein (Figure 1C,D). Moreover, the V5-probed blots also showed significantly increased cleavage fragments at ~30 and ~40 kDa for both smoker and vaper-exposed cells (Figure 1D).

### 2.2. Vapers and Smokers BALF Increase ENaC Activity in Cultured Airway Epithelia

**Figure 2:**
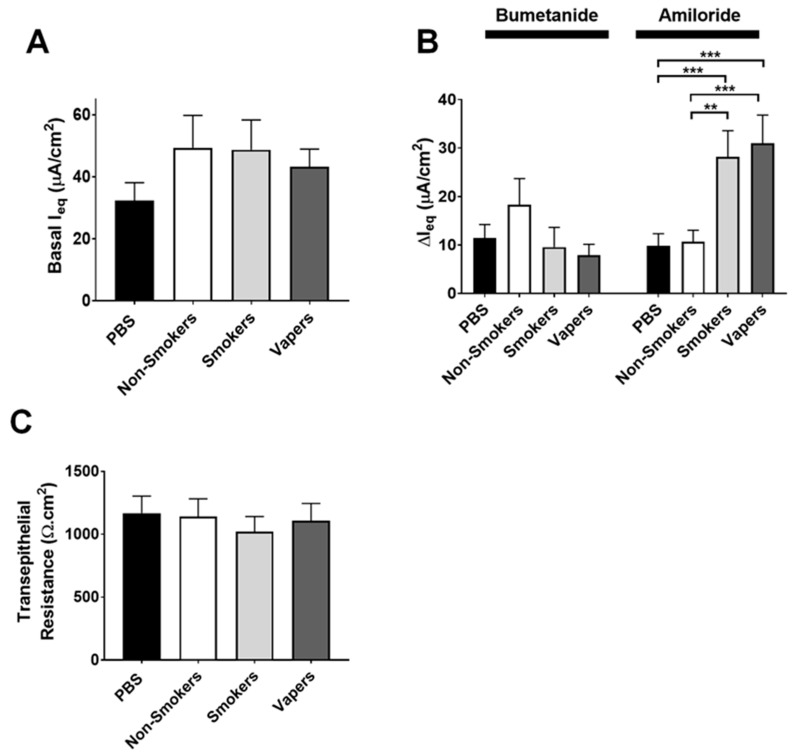
Vapers’ and smokers’ BALF increase ENaC activity. HBECs were exposed to 15 μL of pooled BALF supernatants from non-smokers, smokers, and vapers for 6 h. (**A**) Basal, (**B**) bumetanide- and amiloride-sensitive equivalent currents (I_eq_) were determined under thin-film conditions, and (**C**) macroelectrodes were used to determine transepithelial resistance. ** = *p* < 0.01, *** = *p* < 0.001 different as indicated. All n = 18 per group. All data were analyzed by ANOVA followed by Tukey’s post-test.

We next performed electrophysiological measurements to study the impact of vapers’ airway secretions on ENaC activity in HBECs. We used BALF samples from the same vaper and smoker subjects that had previously been shown to have elevated protease levels, as well as BALF from the same healthy non-users with low protease levels [21,25] and added these to HBECs derived from healthy normal donors to evaluate their effects on airway ion transport. Following 6 h of BALF exposure to HBEC mucosal surfaces, we measured the equivalent current (I_eq_) as indicated in the Methods. The baseline I_eq_ was not significantly different across groups (Figure 2A). We have previously demonstrated that serosal bumetanide inhibits CFTR-mediated transepithelial Cl^−^ secretion in HBECs from normal donors and that this response is absent from HBECs derived from CF donors with severe CFTR mutations [26]. Similarly, we have shown that amiloride added mucosally after the addition of bumetanide can accurately measure transepithelial voltages caused by ENaC-led Na^+^ absorption [26]. Accordingly, we then sequentially added serosal bumetanide, followed by mucosal amiloride, to measure the relative contributions of CFTR-mediated transepithelial Cl^−^ secretion and ENaC-led Na^+^ absorption, respectively. Bumetanide significantly inhibited I_eq_ across all groups by ~10 mA.cm^2^, indicating that Cl^−^ secretion was not affected by BALF exposure (Figure 2B). In contrast, the amiloride-sensitive I_eq_ was significantly elevated after the addition of both vapers’ and smokers’ BALF compared to either non-smokers’ BALF or phosphate-buffered saline (PBS) (Figure 2B), most likely indicating that ENaC activity had increased. The transepithelial resistance was unaltered by BALF exposures (Figure 2C), indicating that epithelial integrity was maintained throughout the series of experiments.

### 2.3. Proteases in Vapers’ and Smokers’ BALF Cause ASL Dehydration

**Figure 3:**
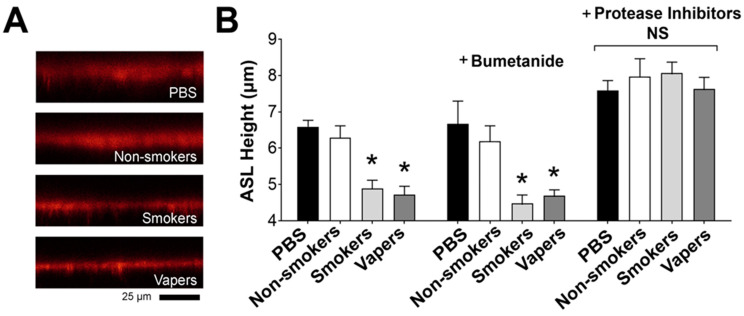
Vapers’ and smokers’ BALF induce airway dehydration. (**A**) Representative XZ-confocal micrographs of ASL (red) after 6 h of mucosal exposure to 15 μL PBS or BALF supernatant labeled with 0.1 mg/mL tetramethylrhodamine dextran. (**B**) Bar graph of 6 h ASL heights following exposure to mucosal BALF ± serosal bumetanide (10 mM) or BALF with mucosal protease inhibitor cocktail. * = *p* < 0.05 different from control. All n = 9 per group. All data were analyzed using the Kruskal–Wallis Test followed by Dunn’s Multiple Comparison post-test.

When PBS is deposited on HBEC mucosal surfaces, excess ASL is absorbed via an ENaC-led process. This absorptive process is sensitive to proteolysis, and increased protease levels lead to ASL dehydration via increased ENaC activity [11]. We hypothesized that the elevated protease levels seen in cigarette and e-cigarette users’ lungs [21,22] caused the increased ENaC activity seen in Figure 1 and Figure 2 and would lead to ASL volume dehydration. Accordingly, we exposed well-differentiated HBECs to BALF supernatants and measured ASL height by confocal microscopy. As with the Western blotting and electrophysiological studies, BALF from non-smokers did not differ from the PBS control (Figure 3A,B). By contrast, both smokers’ and vapers’ BALF significantly decreased ASL height compared to the controls (Figure 3A,B). We next assessed whether altered Cl^−^ secretion contributed to this dehydration. The addition of bumetanide to the serosal media did not alter the relative pattern of ASL responses, and ASL dehydration induced by smokers’ and vapers’ BALF persisted in the absence of Cl^−^ secretion. Interestingly, a cocktail of protease inhibitors fully attenuated the decreases in ASL height induced by either smokers’ or vapers’ BALF (Figure 3A,B), indicating that the ASL dehydration was protease-mediated.

### 2.4. E-Liquids Increase Protease Release and Can Indirectly Cause ASL Dehydration

**Figure 4:**
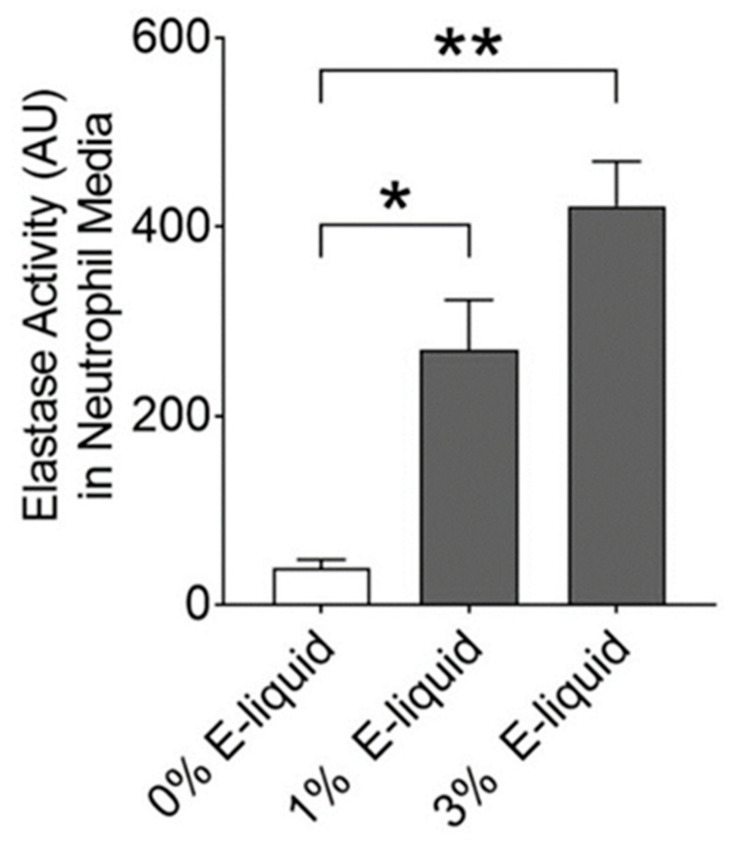
Juul e-cigarette condensate induces protease release from neutrophils. Peripheral blood neutrophils were exposed to vehicle (0% e-liquid) vs. 1% and 3% Juul menthol e-liquids diluted in media for 2 h, and the ANS was collected. Neutrophil elastase activity was determined by measuring the cleavage of a fluorogenic substrate by plate reader. The bar graph shows neutrophil elastase activity in ANS treated as indicated. * = *p* < 0.05, ** = *p* < 0.01 different as indicated. All n = 6 per group. All data were analyzed using the Kruskal–Wallis Test followed by Dunn’s Multiple Comparison post-test.

We have previously demonstrated that nicotine can induce protease release from alveolar macrophages and peripheral blood neutrophils [21]. To characterize the impact of e-liquid-induced protease release from neutrophils on ENaC regulation, we exposed healthy human peripheral blood neutrophils to 1% and 3% Juul menthol e-liquid for 2 h, then collected and processed the media to obtain activated neutrophil supernatant (ANS). Exposure to 1% and 3% e-liquid, but not the vehicle (0% e-liquid) caused a significant increase in neutrophil elastase activity, indicating that e-liquid exposure had triggered neutrophil degranulation/protease secretion (Figure 4).

**Figure 5:**
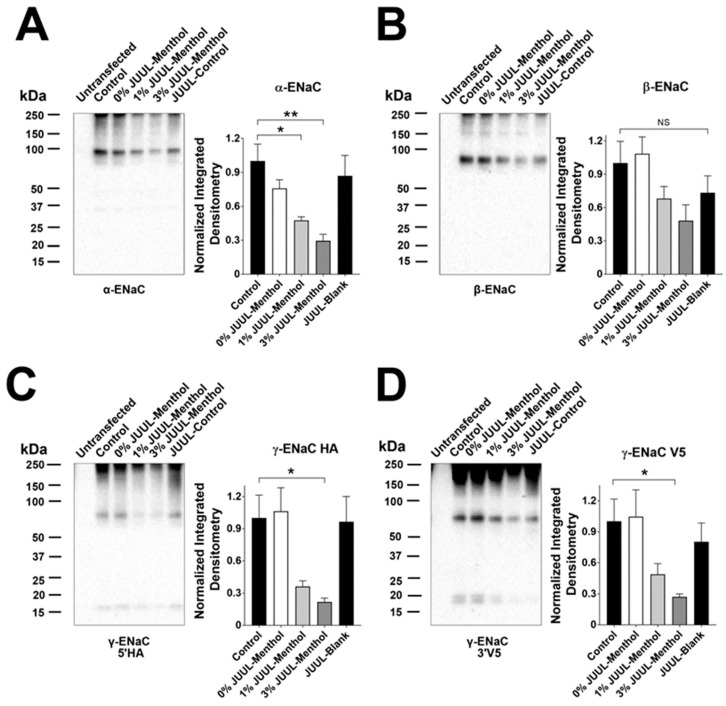
ANS from e-liquid-exposed neutrophils decreases total ENaC levels. ANS from control (naïve media), vehicle (ANS, 0% e-liquid) or Juul menthol e-liquid-treated neutrophils (1% and 3%) were added to ENaC-expressing HEK293T cells for 1 h. (**A**–**D**) Western blots and bar graphs of integrated densitometry for ENaC subunits as indicated. * = *p* < 0.05, ** = *p* < 0.01, different as indicated. All n = 4. All data were analyzed using the Kruskal–Wallis Test followed by Dunn’s Multiple Comparison post-test.

We then exposed α-gfp, β, and HA-γ-V5 ENaC-transfected HEK293T cells to ANS and monitored changes in ENaC by Western blotting. Incubation with e-liquid-treated ANS reduced the level of full-length α-ENaC (Figure 5A). Only ANS from neutrophils exposed to 3% e-liquid reduced the amount of full-length β-ENaC (Figure 5B). Both 1 and 3% e-liquid-exposed ANS reduced 5′-HA and 3′-V5 γ-ENaCs (Figure 5C,D). Interestingly, neither vehicle-exposed neutrophil supernatant nor direct addition of Juul e-liquid altered α, β and γ ENaC subunit size, further indicating that these events are protease-mediated. GAPDH loading controls are shown in Supplemental Figure S2C.

**Figure 6:**
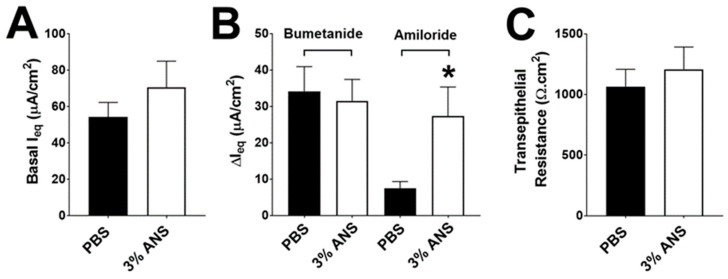
ANS from e-liquid-exposed neutrophils upregulates ENaC activity. HBECs were exposed to 15 μL of PBS or ANS for 6 h. (**A**) Basal and (**B**) Bumetanide- and amiloride-sensitive equivalent currents (I_eq_s) were determined under thin-film conditions. (**C**) Macroelectrodes were used to determine transepithelial resistance. * = *p* < 0.05 different per group as indicated. All n = 12 per group. All data were analyzed with a Student’s *t*-test.

Since e-liquid exposure increased neutrophil elastase secretion and cleaved ENaC (Figure 4 and Figure 5), we then explored the interaction between e-liquid-induced ANS and HBEC ion/fluid transport. ANS from 3% Juul-exposed neutrophils had no effect on baseline I_eq_ (Figure 6A) nor on bumetanide-sensitive I_eq_ (Figure 6B). However, consistent with effects on ENaC subunits expressed in HEK293T cells (Figure 5), 3% Juul-ANS significantly increased the amiloride-sensitive I_eq_, indicating that ENaC activity had increased (Figure 6B). Transepithelial resistance was not altered by the addition of ANS from 3% e-liquid-exposed neutrophils, indicating that the change in ion transport was not due to altered epithelial barrier properties (Figure 6C).

**Figure 7:**
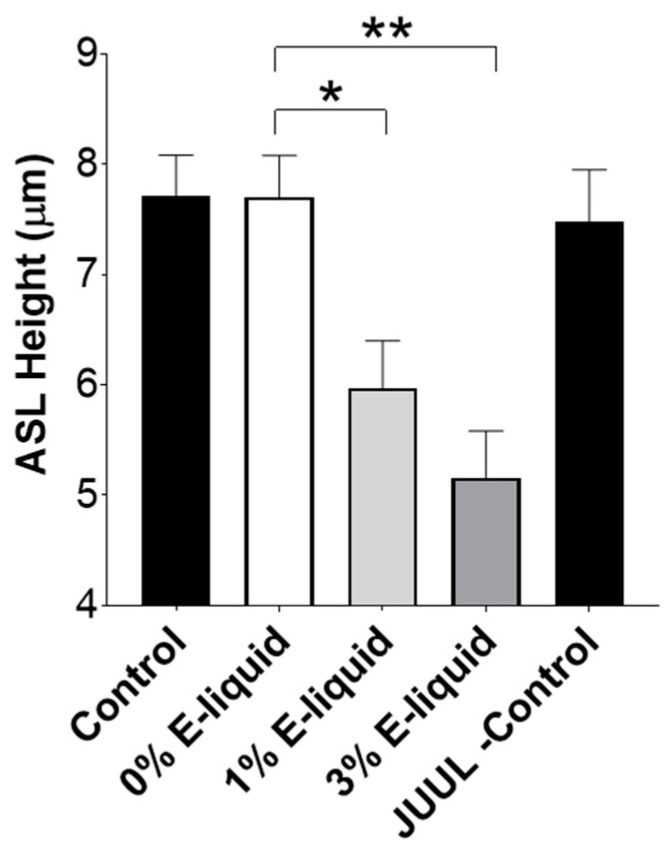
ANS from Juul-exposed neutrophils induces airway dehydration. HBECs were exposed mucosally to 15 μL of PBS (control), naïve ANS (0% e-liquid), ANS following 1–3% e-liquid exposure or 3% e-liquid in PBS, all labeled with 0.1 mg/mL tetramethylrhodamine-dextran and imaged by XZ-confocal microscopy 6 h later. The bar graph shows 6 h ASL heights. * = *p* < 0.05, ** = *p* < 0.01 different as indicated. All n = 6 per group. All data were analyzed using the Kruskal–Wallis Test followed by Dunn’s Multiple Comparison post-test.

Finally, to explore the possibility that increased proteases in ANS post-e-liquid exposure induced airway dehydration, we exposed HBEC mucosal surfaces to ANS and monitored ASL height. ANS from e-liquid-exposed neutrophils caused significant decreases in ASL height compared to the untreated (0%) neutrophil media (Figure 7). Consistent with the Western blots (Figure 5), neither the media control nor direct 3% e-liquid exposure affected ASL height (Figure 7).

## 3. Discussion

Cleavage by intracellular, membrane-bound and extracellular proteases determines ENaC’s activation state [27]. The cleavage and release of an inhibitory peptide from the extracellular domains of α- and γ- subunits changes the conformation of the channel, increasing the open probability and allowing Na^+^ conduction. Due to the prevailing electrochemical gradients in the airways, increased ENaC activity leads to Na^+^ absorption, with concomitant paracellular movement of Cl^−^ and transepithelial water flow through aquaporins and paracellularly [11]. Incidentally, increased proteolysis of ENaC and the subsequent Na^+^ hyperabsorption/ASL dehydration have been proposed as contributing factors for CF. Similarly, overexpression of βENaC in mice lungs leads to airway dehydration, mucus plugging, inflammation and increased mortality, which is similar to the phenotype seen in CF and COPD patients [28]. Thus, increased ENaC activity is a validated hydration-related biomarker of harm. Consistent with previous studies [29], we found that neutrophil elastase increased cleavage of γ-ENaC (Figure S1). While we detected γ-ENaC cleavage fragments after the addition of BALF, we inconsistently detected cleavage of γENaC or other subunits following either BALF or ANS addition (Figure 1A,B and Figure 5A–D). In vitro proteolytic cleavage studies with ENaC are usually performed using low levels of single-species proteases (e.g., 100 nm neutrophil elastase) [20]. In contrast, we do not know what concentrations of proteases are present in our biological samples, and possibly much higher levels than 100 nM may have been present. Additionally, our samples likely contained multiple proteases. For example, we know that neutrophil elastase, matrix metalloprotease-2 and -9 were upregulated in vapers’ BALF [21], and other proteases may also have been upregulated. Thus, while exposure of HBECs to multiple proteases absolutely resulted in increased amiloride-sensitive currents (Figure 2B and Figure 6B), the cleavage fragments may have been degraded either by high levels of proteases and/or multiple proteases to the point that they were no longer detectable by Western blotting. Moreover, fragments of α- and γ- ENaC are released into the ASL by cleavage [30]—if these fragments are detectable in BALF or sputum, they may constitute a novel biomarker of ENaC activity in human vapers and smokers. 

Ion and water movement have been extensively studied in the airways of combustible tobacco smokers. For example, both acute and chronic smoking reduce CFTR-mediated Cl^−^ secretion and slow mucus transport in humans [31,32]. Similarly, in vitro combustible tobacco exposures decreased CFTR activity and induced ASL dehydration [32,33]. Moreover, mucus dehydration, which is typically caused by ion transport dysfunction, inversely correlates with FEV_1_ in COPD patients [12]. To date, the impact of vaping on ion and fluid transport has not been studied in vivo in vapers. However, acute reductions in CFTR-mediated ion transport in HBECs exposed to e-cigarette vapor have been reported [34], and Kim et al. found that e-cigarette exposure inhibited ATP-sensitive K^+^ channels (so-called big potassium or BK channels), leading to ASL dehydration and mucus stasis [35]. These studies differed from our current study in that they directly exposed HBECs to cigarette smoke or e-cigarette vapor, while we added airway secretions from smokers and vapers to HBECs. However, both methodological approaches show impaired ion transport in HBECs after both cigarette smoke and e-cigarette smoke exposure. We did not look for altered K^+^ channel activity and further studies will be required to determine the full extent of ion channel dysfunction in vapers.

HBECs have been extensively used to study CF lung disease. Indeed, the ion and fluid transport dysfunction in CF HBECs matches what is seen in CF patients very well (i.e., both HBECs and CF airways show ASL dehydration and mucus stasis) [36,37]. A key aspect of our electrophysiological studies is that we are measuring changes in transepithelial voltages, which require functional channels and transporters on both apical and basolateral membranes [38]. An additional consideration is that we are studying transepithelial ion transport under open circuit conditions with native ASL present, which limits the test compound that can be applied mucosally. Indeed, the addition of PBS solution to the mucosal surface alters amiloride-sensitive Na^+^ absorption [39,40]. We therefore add amiloride as a dry powder, which rapidly dissolves in the ASL to inhibit ENaC and prevent unwanted fluid-induced changes in transepithelial voltages that would occur if amiloride was added in a liquid vehicle. Since we are measuring transepithelial ion transport under open circuit conditions, if we add amiloride first, we will hyperpolarize the apical membrane and induce Cl^−^ secretion [38]. Thus, we add bumetanide first to isolate the change in voltage caused by Cl^−^ secretion, followed by the addition of amiloride to measure the voltage caused by Na^+^ absorption. Using this approach, we can pick up significant bumetanide-sensitive transepithelial voltages in normal but not CF HBECs and a significantly greater amiloride-sensitive voltage in CF relative to normal HBECs [26,39]. We acknowledge that bumetanide has the potential to inhibit both CFTR and Ca^2+^-activated Cl^−^ secretion. However, under the conditions tested, the Ca^2+^-activated Cl^−^ secretion pathway is quiescent due to the lack of the natural agonist (e.g., ATP) [26], so we are confident that with serosal bumetanide, we are measuring CFTR-mediated (transepithelial) Cl^−^ secretion.

Similarly, as discussed above, changes in ion transport seen in HBECs are identical to what is seen in smokers [32]. Interestingly, both acute and chronic cigarette smoke exposures result in ASL dehydration on HBEC surfaces that is caused by smoke-induced diminution of CFTR [32,41]. Thus, their use for probing potential e-cigarette-induced ion and fluid dysfunction is appropriate. We have previously added normal and CF sputum to HBEC mucosal surfaces, and we found that CF sputum induces airway dehydration due to increased proteolytic activity [42]. Our data indicated that smokers’ BALF caused airway dehydration (Figure 3). This is consistent with the dehydrated airway secretions detected in smokers’ lungs [32], suggesting that our approach of adding BALF to HBECs and measuring ion and fluid transport is valid. We detected dehydration after exposure of vapers’ BALF to HBECs (Figure 3), and based on the similarities of HBEC ion/fluid responses to CF patients and smokers, it is likely that ASL dehydration/mucus stasis will also occur in vapers. This is potentially very serious since mucus stasis will (i) eventually lead to occlusion of the small airways and decreased gas exchange across the alveoli and (ii) promote bacterial infections and inflammation. Thus, based on our data, we recommend that cohorts of chronic vapers be assessed for ion transport dysfunction and mucus stasis/dehydration.

Our data indicate that the ASL dehydration seen after exposure to vapers’ and smokers’ BALF was due to increased ENaC-led Na^+^ absorption rather than to reduced CFTR-mediated Cl^−^ secretion. Indeed, pretreating BALF with a cocktail of protease inhibitors before the BALF was added to HBEC mucosal surfaces prevented ASL dehydration (Figure 3). Nicotine can directly inhibit CFTR-mediated Cl^−^ secretion, which would also contribute to ASL volume depletion [33,43], and we cannot exclude the possibility that there is a nicotine-related component to ASL dehydration (i.e., nicotine-mediated protease release from immune cells that activates ENaC; Figure 4, Figure 5, Figure 6 and Figure 7) coupled with direct effects of e-cigarettes or combustible tobacco smoke on the epithelia. Indeed, in humans, both effects may occur, which would potentiate any ASL dehydration. Thus, a potential limitation of our study is that we have only explored the indirect/immune-mediated effects of vaping on ion and fluid transport, and further studies will be needed to better understand the combined effects of vaping and protease release. However, to our knowledge, this is one of the first studies that has looked at how smokers’ and vapers’ airway secretions can impact ion transport.

Persistent neutrophilic infiltration and inflammation are pathological features of several chronic respiratory diseases [44]. Furthermore, smoking and vaping may increase the infiltration of neutrophils in the users’ lungs [45,46]. Hence, we explored the possibility that e-liquid exposure to neutrophils can cause protease release and subsequent ENaC activation. Indeed, relevant levels of e-liquids (1% and 3%) caused neutrophil elastase release from peripheral blood neutrophils (Figure 4 and Figure 5A). When HBECs were exposed to ANS, we observed significant increases in amiloride-sensitive I_eq_, but not bumetanide-sensitive I_eq_ (Figure 6) and a concomitant decrease in ASL height (Figure 7), confirming the potential for e-liquid-mediated protease release to dehydrate mucosal surfaces by altering Na^+^ absorption.

We understand that our study has several limitations. First, although we have used age- and sex-matched subjects in each group in our study, we used pooled BALF supernatants, and it would be interesting to see how individual subjects induced ASL dehydration. Second, to more precisely determine ENaC subunit cleavage, we used epitope-tagged ENaC subunits transfected into HEK293T cells instead of studying endogenous channels, which may show different cleavage patterns. Finally, apart from neutrophils, other cell types, such as alveolar macrophages, may also release proteases, which should be further investigated to obtain a complete picture of vaping-mediated effects on airway hydration.

In summary, we have found that BALF from age- and sex-matched smokers and vapers with known increases in proteolytic activity affects ENaC subunit densities, activates amiloride-sensitive I_eq_s, and drives ASL dehydration in a protease-sensitive manner. Similarly, relevant concentrations of e-liquids induce neutrophil degranulation, and supernatant (ANS) from these e-liquid-exposed neutrophils also increases amiloride-sensitive I_eq_ and ASL absorption. We conclude that proteases in smokers’ and vapers’ lungs activate ENaC, leading to increased Na^+^ absorption and airway dehydration. If this persists for extended periods, as is expected with chronic vaping or smoking, this may result in dehydration/ mucus accumulation, infection and development of lung disease. Hence, given the potential harmful health effects associated with the long-term use of e-cigarettes, claims about their safety should be reconsidered.

## 4. Materials and Methods

### 4.1. Subject Recruitment and Sample Collection

Details of the study design and the subject recruitment criteria have been published [21]. The University of North Carolina at Chapel Hill, School of Medicine Committee on the Protection of the Rights of Human Subjects approved the study protocol (#13-2227). Bronchoalveolar lavage fluids (BALF) were obtained using bronchoscopy as described [21]. Equal volumes of pooled BALFs from age and sex-matched subjects of non-smokers, smokers, and vapers were concentrated using 3 kDa centrifugal filter units (Amicon Ultra-2, MilliporeSigma Lenexa, KS, USA, cat no. UFC200324) following manufacturer’s instructions and added directly to cells (Supplementary Table S1).

### 4.2. Cell Culture

HBECs were isolated from donor human lungs as described previously [47]. The protocol was approved by the UNC Biomedical Institutional Review Board (#03–1396). Passage 1 HBECs from equal numbers of male and female donors were seeded on collagen (MilliporeSigma Lenexa, KS, USA, cat no. C7521)-coated cell culture inserts (24-well, polyester 0.4 µm; Oxyphen, Wetzikon, Switzerland). Cultures were incubated at 37 °C in 5% CO_2_ and changed to air-liquid interface (ALI) after confluence. Cultures were used after ~4 weeks of differentiation. Human embryonic kidney cells (HEK293T) were cultured in Dulbecco’s Modified Eagle Medium (DMEM, MilliporeSigma Lenexa, KS, USA) supplemented with 10% fetal bovine serum (FBS), penicillin/streptomycin (MilliporeSigma, Lenexa, KS, USA) at 37 °C in 5% CO_2_. 

### 4.3. ASL Height Measurements

ASL height was measured as described [48]. Briefly, pooled BALFs or neutrophil supernatants from blood were mixed 10:1 with PBS containing 0.1 mg/mL 10 kDa tetramethylrhodamine-labeled dextran (Thermo Fisher Scientific, Waltham, MA, USA, cat no. D1816) and 15 μL BALF added mucosally per culture. ASL was imaged using either a Leica SP5 or a SP8 confocal microscope in XZ-mode with a 561 nm laser (excitation at 600 ± 20 nm) and a 63X glycerol lens (1.2 NA). Images were collected automatically at 10 pre-determined points per culture after the addition of BALF or PBS. Perfluorocarbon (Thermo Fisher Scientific, Waltham, MA, USA, cat no. 394890500) was added prior to all measurements to prevent evaporation.

### 4.4. Electrophysiological Measurements

HBECs were exposed to pooled 15 μL BALF per culture for 6 h vs. PBS (control). After this time, perfluorocarbon (Thermo Fisher Scientific, Waltham, MA, USA, cat no. 394890500) was added to prevent evaporation and membrane potentials were recorded using a KCl-filled glass microelectrode that was positioned in the ASL with a micromanipulator and a KCl/agar macroelectrode that was positioned in the serosal media. Transepithelial voltages were then recorded using an FD223 electrometer (World Precision Instruments, Sarasota, FL, USA) as described [23,26]. Changes in voltages caused by CFTR-mediated transepithelial Cl^−^ secretion and ENaC-mediated transepithelial Na absorption were determined by calculating bumetanide and amiloride-inhibitable voltages, respectively. We then measured resistance using an Epithelial Volt Ohm Meter (EVOM2, World Precision Instruments, Sarasota, FL, USA) as described [48]. Equivalent open circuit currents (I_eq_s) were then calculated according to Ohm’s law.

### 4.5. Transfection of HEK293T Cells with ENaC Subunits and Western Blotting

HEK293T cells were cultured until they were 80–90% confluent and were transfected using Lipofectamine 2000 (Thermo Fisher Scientific, Waltham, MA, USA, cat no. 11668-027) with a green fluorescent protein (GFP)-labeled α-ENaC, unlabeled β-ENaC and γ-subunit labeled with hemagglutinin (HA) and V5 on the on the 5′ and 3′ termini, respectively. Following 24 h of transfection, expression of ENaC was confirmed by detection of GFP fluorescence. α, β, γ-ENaC-transfected cells were exposed to BALFs, 100 nM human neutrophil elastase [Elastin Products Company, Owensville, MO, USA] or media (control), washed with ice-cold PBS and lysed in RIPA buffer (MilliporeSigma Lenexa, KS, USA cat no. 20-188; 50 mM Tris-HCl, pH 7.4, 150 mM NaCl, 0.25% deoxycholic acid, 1% NP-40, 1 mM EDTA) with 0.1% SDS and protease inhibitors (Roche Diagnostics, Indianapolis, IN, USA, cat no. 11873580001) and stored at −80 °C. Lysate protein concentration was determined using the BCA Protein Assay (Thermo Fisher Scientific, Waltham, MA, USA, cat no. 23225). Protein was resolved on 4–15% MiniProtean^®^ TGX™ precast gels, followed by transfer to PVDF membranes (Bio-Rad Hercules, CA, USA). Membranes were incubated with primary antibodies (α-ENaC, Novus Biologicals, Centennial, CO, USA cat no. NB100-74357; β-ENaC, ABclonal, Woburn, MA, USA cat no. A1765; anti-HA, Cell Signaling Technology, Danvers, MA, USA cat no. 3724; anti-V5, Thermo Fisher Scientific Waltham, MA, USA, cat no. R960-25; GAPDH, Cell Signaling Technology Danvers, MA, USA cat no. 2118), followed by the appropriate secondary antibodies (Jackson ImmunoResearch Laboratories, West Grove, PA, USA). Blots were developed using Clarity western ECL substrate on a ChemiDoc™ MP Imaging system (Bio-Rad, Hercules, CA, USA). Integrated densitometric analysis was performed using NIH ImageJ software (version 1.53s) as described [21], and data were normalized to corresponding GAPDH and to control blots per condition.

### 4.6. Isolation of Neutrophils from Blood and Exposure to Menthol-Flavored E-Liquid

Neutrophils were isolated from human peripheral whole blood [IRB #13-3454] by immune-magnetic negative selection (EasySep™ Direct Human Neutrophil Isolation Kit, STEMCELL Technologies, Vancouver, BC, Canada, cat no. 19666) as described [21]. Freshly isolated neutrophils were re-suspended in 10 mM HEPES (pH 7.6)-buffered RPMI 1640 media. At the time of experimentation, cells were transferred to Ringer’s solution (in mM: 120 NaCl, 5.2 KCl, 1.2 MgCl_2_, 1.2 CaCl_2_.2H_2_O, 12 NaHCO_3_, 24 HEPES, 10 glucose, pH 7.4) and exposed to vehicle vs. 1% or 3% Juul-Menthol e-liquid for 2 h at 37 °C. Juul e-cigarettes were purchased from local vendors in Chapel Hill, NC, USA. Media was centrifuged, and the activated neutrophil supernatants (ANS) were frozen at −80 °C until needed. Neutrophil elastase activity in naïve media or ANS was evaluated by monitoring the cleavage of a neutrophil elastase-specific fluorogenic peptide (Suc-Ala-Ala-Ala-MCA, Peptides International, Louisville, KY, USA) using an M1000 multi-plate reader (Tecan, Raleigh, NC, USA) as described [21]. 

### 4.7. Statistical Analysis

All datasets were checked for normality, and parametric or non-parametric analyses were selected as appropriate. Pairwise comparisons were determined using Student’s t-test or the Mann-Whitney-U test. Multiple comparisons were determined using ANOVA followed by Tukey’s post-test or the Kruskal–Wallis test followed by Dunn’s Multiple Comparison test as appropriate. *p*-values < 0.05 were considered significant. Experiments were repeated at least three times. All data are shown as mean ± SEM.

## Data Availability

The original data is available to researchers upon request.

## Supplementary Materials

The following supporting information can be downloaded at: https://www.mdpi.com/article/10.3390/ijms242015348/s1.
