# Supplementary material for: Vaping-Induced Proteolysis Causes Airway Surface Dehydration
Source: Int J Mol Sci. 2023 Oct 19;24(20):15348. doi: 10.3390/ijms242015348 (PMC10607227; doi:10.3390/ijms242015348)
Supplement: Supplementary file 1 [file ijms-24-15348-s001.zip › ijms-2627602-supplementary.pdf]

	Non-smokers	Smokers	Vapers
**Ethnicity (African American: Caucasian)**	1:3	2:2	0:4
**Female/Male**	2/2	2/2	2/2
**Age**	31.25 ± 8.26	39.5 ± 8.23	26.75 ± 7.68
**BMI**	23.95 ± 2.39	35.90 ± 6.57	32.13 ± 10.00
**Forced Vital Capacity (FVC) (%)**	99.25 ± 14.03	101.75 ± 18.86	109.00 ± 4.08
**Forced Expiratory Volume in 1 second (FEV1) (%)**	90.75 ± 4.43	98.5 ± 17.08	107.5 ± 8.54

Supplementary Table S1 Demographics details of the subjects used in the study. Values are represented as mean ± SD. FVC and FEV_1 are shown as "percent predicted" and were compared to predicated values based on age, sex and weight, following American Thoracic Society and European Respiratory Society guidelines.

Supplementary Figures

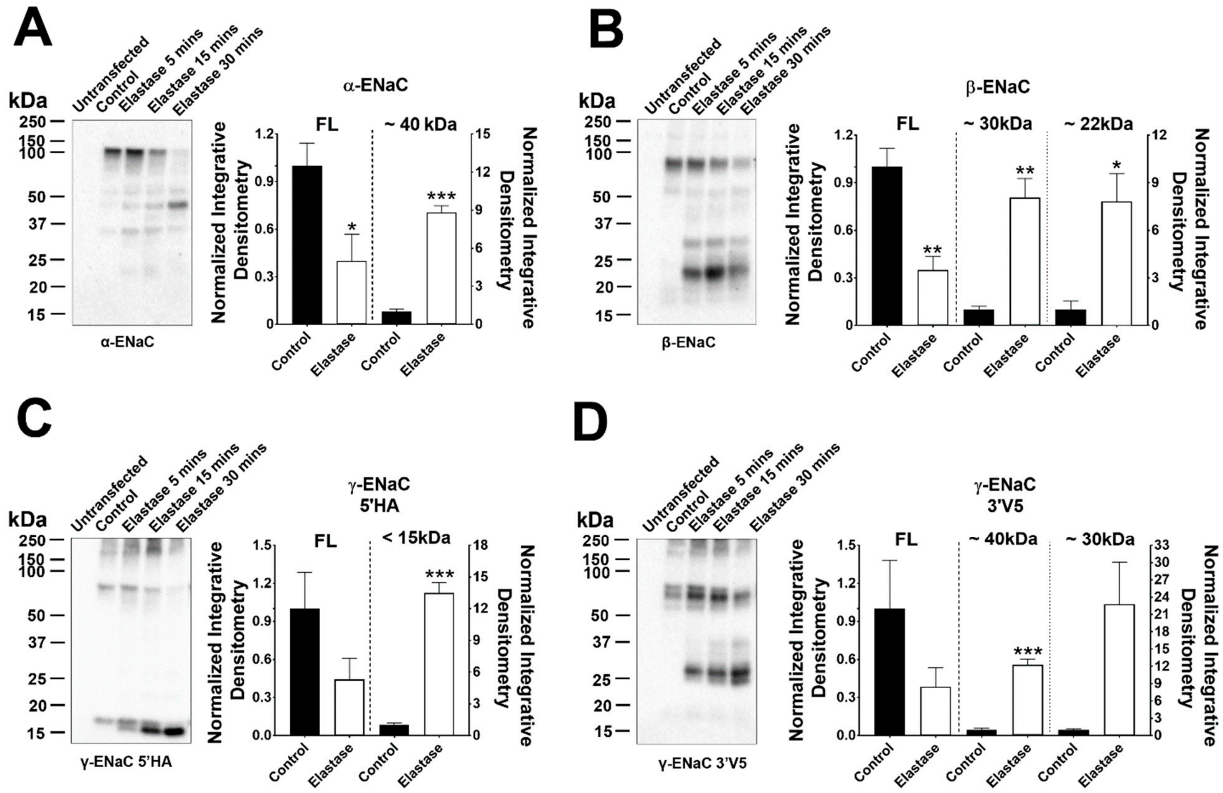

Supplementary Figure S1. HEK293T cells expressing α-ENaC-gfp, β-ENaC, γ-HA-ENaC-V5 subunits were treated with 100 nM of elastase protease for 5, 15 and 30 min, and lysate was collected for Western blots. Elastase caused degradation of full-length A) α–, B) β, C) 5'HA-γ and D) 3'V5-γ subunits in a time-dependent manner. Integrated densitometric analysis of control versus 30 min post-elastase treatment sample is shown as bar graphs for both full length subunits and the cleavage products. Data represented as mean ± SEM, *= $p<0.05$, ***= $p<0.001$. All n=4 per group. All data were analyzed using the Kruskal-Wallis test followed by Dunn's Multiple Comparison post-test.

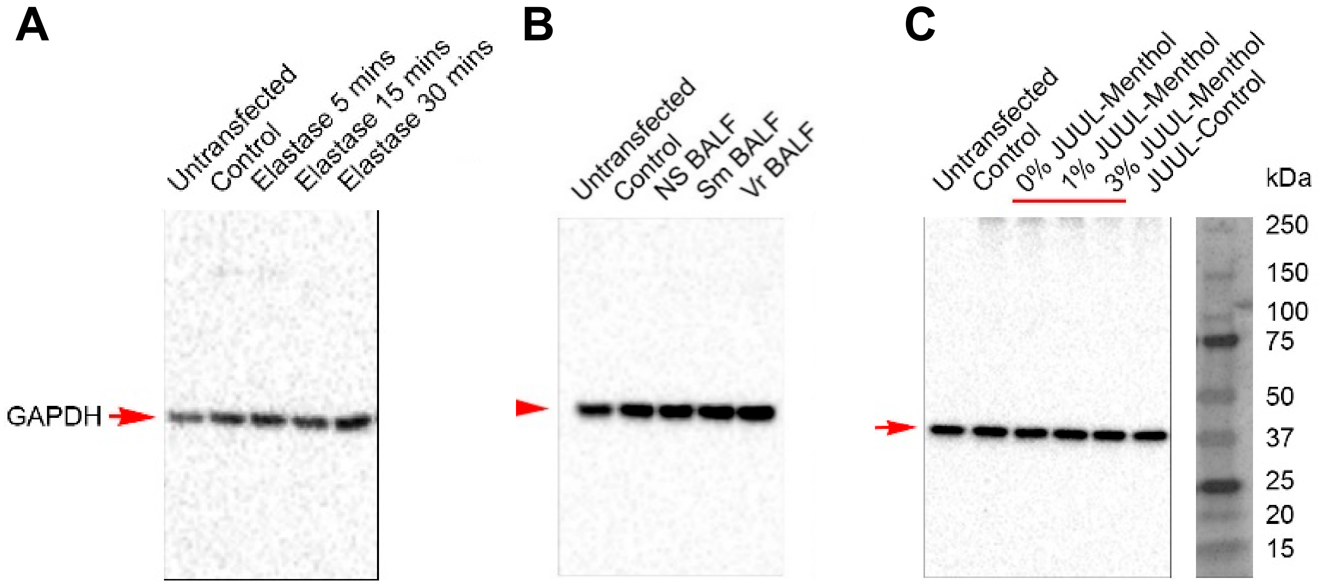

Supplementary Figure S2. Representative GAPDH loading controls for all Western blots. A, figure S1; B; Figure 1, C, Figure 5.
